# Prevalence of cardiac morphological and functional alterations in systemic lupus erythematosus patients with a low disease activity

**DOI:** 10.1186/1532-429X-15-S1-P94

**Published:** 2013-01-30

**Authors:** Florian Andre, Maria Fernanda Braggion Santos, Hassan Abdel-Aty

**Affiliations:** 1 Department of Cardiology, University of Heidelberg, Heidelberg, Germany; 2 School of Medicine of Ribeirao Preto University of Sao Paulo, Sao Paulo, Brazil

## Background

Cardiac involvement in systemic lupus erythematosus (SLE) is a common complication and is associated with a considerable morbidity and mortality in these patients. As affected individuals often present with subclinical or non-specific symptoms the betimes confirmation of cardiac manifestations is challenging. Cardiovascular magnetic resonance (CMR) with late gadolinium enhancement (LGE) is the current gold-standard for non-invasive tissue characterization as well as for the evaluation of systolic and diastolic function. In this study we assessed the cardiac morphology and function in SLE patients with low disease activity.

## Methods

We studied twenty-nine SLE patients (3 male, 26 female) fulfilling the SLE diagnostic criteria of the American College of Rheumatology and compared them to thirty age-matched healthy volunteers. All patients were in a stable clinical condition and only patients with a low disease activity (SLEDAI Index < 5) were included. All of them received an individually optimized medication. CMR images were acquired on a 1.5 T whole-body MRI (Achieva, Philips Healthcare, Best, The Netherlands) using a 32-element cardiac phased-array coil. Short and long axis views were obtained applying a standard clinical steady-state free-precession sequence and LGE CMR imaging was performed (Gadolinium-DTPA 0.2 mmol/kg body weight, Magnevist, Schering, Berlin, Germany) in 27 patients. Left and right ventricular (LV, RV) end-diastolic and end-systolic volumes (EDV, ESV), stroke volume (SV), ejection fraction (EF) as well as the mitral/tricuspid annular plane systolic excursion (M/TAPSE) and LV wall mass were measured.

## Results

SLE patients and healthy controls had comparable demographic and clinical characteristics. SLE patients had significantly higher LV-EDV, LV-ESV and LV-EF. LV-SV and MAPSE did not differ significantly between patients and healthy controls. However SLE patients had significantly higher RV-EDV, RV-ESV, RV-SV, RV-EF and TAPSE values. LV mass showed no significant differences. Nineteen of twenty-seven assessed patients (70%) showed LGE CMR as a sign of myocardial involvement.

## Conclusions

Even in SLE patients with a low disease activity there is a high prevalence of myocardial and functional alterations. Especially the RV function is affected which could be due to early onset pulmonary manifestations of SLE. Regarding the high morbidity and mortality of cardiac complications in SLE patients, CMR may be an important non-invasive method for the early diagnosis of cardiopulmonary involvement.

## Funding

none

**Table 1:** Comparison of SLE patients to healthy controls

	SLE patients (n=29)	Healthy controls (n=30)	
LV EF (%)	64.1±9.4	68.4±5.4	p<0.05
LV EDV (ml)	152.7±31.0	129.1±30.9	p<0.01
LV ESV (ml)	56.3±24.3	41.7±16.0	p<0.01
LV SV (ml)	96.5±19.5	87.3±16.8	p=n.s.
LV Cardiac output (l/min)	7.0±1.9	6.1±1.2	p<0.05
MAPSE (mm)	12.9±2.7	14.6±4.1	p=n.s.
LV EDV/BSA (ml/m^2^)	85.7±17.5	73.6±14.2	p<0.01
LV ESV/BSA (ml/m^2^)	31.8±15.2	23.8±8.3	p<0.05
LV SV/BSA (ml/m^2^)	53.9±9.6	49.8±7.0	p=n.s.

RV EF (%)	52.9±9.0	60.2±7.4	p<0.01
RV EDV (ml)	178.0±29.5	127.1±34.6	p<0.0001
RV ESV (ml)	83.8±20.6	50.8±17.3	p<0.0001
RV SV (ml)	94.2±24.0	76.3±21.2	p<0.01
RV Cardiac output (l/min)	6.8±2.1	5.3±1.6	p<0.01
TAPSE (mm)	23.3±6.0	20.1±4.7	p<0.05
RV EDV/BSA (ml/m^2^)	52.5±11.5	43.5±10.9	p<0.01
RV ESV/BSA (ml/m^2^)	47.2±12.7	28.9±8.8	p<0.0001
RV SV/BSA (ml/m^2^)	52.5±11.5	43.5±10.9	p<0.001

Wall mass (g)	73.1±19.2	63.3±20.9	p=n.s.
Wall mass/BSA (g/m^2^)	40.7±9.0	35.9±9.5	p=n.s.

**Table 2:** Demographic and clinical characteristics

	SLE patients (n=29)	Healthy controls (n=30)	
Age (years)	44.7±13.4	44.7±11.1	p=n.s
Sex (female)	26/29 (89.7%)	27/30 (90.0%)	p=n.s
Weight (kg)	71.6±15.2	66.8±11.2	p=n.s
BMI (kg/m^2^)	25.9±4.7	23.9±4.0	p=n.s
BSA (m^2^)	1.79±0.19	1.74±0.15	p=n.s
Heart rate (bpm)	70.9±12.6	66.5±9.5	p=n.s
Blood pressure systolic (mmHg)	122.7±10.7	123.8±15.8	p=n.s
Blood pressure diastolic (mmHg)	74.2±11.5	75.6±6.9	p=n.s

